# A Simple Approach to Produce Tailor-Made Chitosans with Specific Degrees of Acetylation and Molecular Weights

**DOI:** 10.3390/polym13152415

**Published:** 2021-07-22

**Authors:** Luis-Felipe Sánchez, Jimmy Cánepa, Suyeon Kim, Javier Nakamatsu

**Affiliations:** 1 Science Department, Pontificia Universidad Catolica del Peru—PUCP, Av. Universitaria 1801, Lima 32, Peru; lsanchezz@pucp.pe (L.-F.S.); j.canepa@pucp.pe (J.C.); 2 Engineering Department, Pontificia Universidad Catolica del Peru—PUCP, Av. Universitaria 1801, Lima 32, Peru; skim@pucp.pe

**Keywords:** chitosan, tailor-made polymers, degree of acetylation, molecular weight reduction, biopolymers

## Abstract

Chitin is a structural polysaccharide that is found in crustaceans, insects, fungi and some yeasts. Chitin deacetylation produces chitosan, a well-studied biopolymer with reported chemical and biological properties for diverse potential applications for drug delivery, metal ion absorption, scaffolds and tissue engineering. Most known properties of chitosan have been determined from samples obtained from a variety of sources and in different conditions, this is, from chitosans with a wide range of degrees of N-acetylation (DA) and molecular weight (MW). However, as for any copolymer, the physicochemical and mechanical characteristics of chitosan highly depend on their monomer composition (DA) and chain size (MW). This work presents a simple methodology to produce chitosans with specific and predictive DA and MW. Reaction with acetic anhydride proved to be an efficient method to control the acetylation of chitosan, DAs between 10.6% and 50.6% were reproducibly obtained. In addition to this, MWs of chitosan chains were reduced in a controlled manner in two ways, by ultrasound and by acidic hydrolysis at different temperatures, samples with MWs between 130 kDa and 1300 kDa were obtained. DAs were determined by ^1^H-NMR and MWs by gel permeation chromatography.

## 1. Introduction

**Figure 1:**
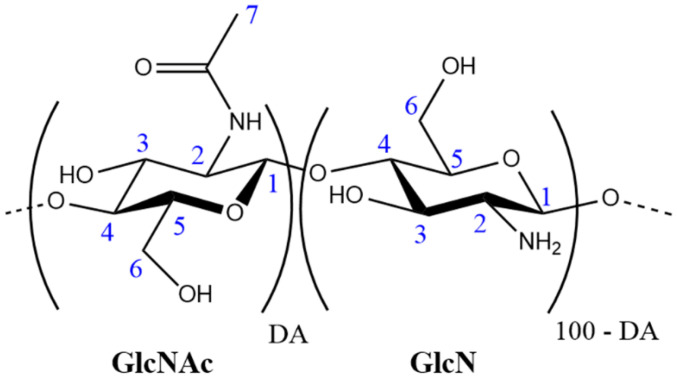
Chitin and chitosan structures showing the acetylated (GlcNAc) and deacetylated (GlcN) units. DA represents the degree of N-acetylation, for chitin DA = 100.

Chitin is a structural biopolymer that is found in some marine invertebrates, insects, fungi and yeasts [1], it is a linear homopolymer composed of N-acetylated glucosamine repeat units and it is the second most abundant polysaccharide in nature [2]. Chitosan is produced from the full or partial deacetylation of chitin, it is a linear polymer formed by (1→4) linked glucosamine and N-acetyl glucosamine units, as shown in Figure 1 [3]. Chitosan is generally soluble in dilute aqueous acidic solutions, which allows it to be purified by pH changes and to produce a myriad of forms such as gels, films, porous membranes, filaments, pellets and microparticles by evaporation or precipitation techniques [4]. In this regard, chitosan has been extensively studied for its potential in applications like drug delivery [5], water purification and heavy metal absorption [6], materials and coatings with antibacterial activity [7], hydrophilic and porous membranes [8], and scaffolds for tissue engineering [9].

The fundamental parameters that determine the physicochemical properties and behavior of chitosan are the degree of N-acetylation (DA), which reflects the proportion of both repeat units, and the molecular weight (MW). As mentioned before, chitin is obtained from different natural sources, besides washing and sorting out impurities, the extraction of chitin requires, demineralization and deproteinization steps [10,11]. Finally, the conversion of chitin to chitosan, the deacetylation process, usually requires harsh reaction conditions such as a high alkali concentration, high temperatures and long reaction times. A wide range of processing parameters for chitosan production has been reported in the literature, each resulting in products with a particular DA and MW, that can differ even from batch to batch [12,13,14,15].

As for any polymer, the size of the chains affects the properties and behavior of the material, for instance, its MW may not only affect the mechanical properties and the viscosity of its solutions, but also its solubility, thermal transitions and crystallinity. In the same way, the chemical composition of a copolymer such as chitosan (characterized by its DA) also affects its solubility, crystallinity and thermal behavior, it can even change its affinity to polar or non-polar solvents and its liquid or gas permeability [16,17]. Moreover, it is known that the conformations of chitosan chains depend strongly on the ionic strength of the solvent [18]. Thus, it is important that the DA and MW of every chitosan sample studied are known. Properties of chitosan such as solubility, gas permeability, viscosity and water contact angles on films have been reported showing a whole range of data, which occurs because of the variety of the samples used, with very different MW and DA. This study reports a simple way to produce tailor-made chitosans: polymer chains with a determined MW and DA. Chitosans with a whole range of DAs were selectively obtained by controlled acetylation with acetic anhydride and, the chains were shortened to a particular size either by acidic hydrolysis at different temperatures or by several short exposures to ultrasound radiation.

## 2. Materials and Methods

### 2.1. Materials

Anhydrous acetic acid (HOAc), sodium acetate trihydrate (NaOAc·3H_2_O), concentrated ammonia, deuterium oxide (>99.8%) and deuterium chloride (>99%) were purchased from Sigma Aldrich and were of reaction grade. Acetic anhydride (Ac_2_O), reaction grade, was purchased from Riedel-de-Haën. Pullulan standards (PSS-pulkit, M_p_ 9.6–2350 kDa) were purchased from PSS. Milli-Q water was used to prepare the GPC buffer solutions and deionized water was used in the acetylation reactions. Highly deacetylated chitosan flakes (weight-average MW = 1206 kDa, DA = 10.6%), labeled Q10, was obtained from the “Modificación de Materiales” research group at PUCP and chitosan powder (weight-average MW = 1632 kDa, DA = 24.3%), labeled Q24, was purchased from Sigma Aldrich.

### 2.2. Characterization of Chitosan

The most common methods to determine the degree of acetylation (or deacetylation) of chitosan are IR and ^1^H-NMR spectroscopies. The most reliable is ^1^H-NMR [19], which can also allow to determine the distribution of the repeat units along the chain [20], however, this method requires that samples be soluble. Solid samples can be characterized by IR spectroscopy, in this case DA determination is a semi-empirical method that compares the relative intensity of different absorption bands.

#### 2.2.1. Determination of the Degree of N-Acetylation (DA) of Chitosan by ^1^H NMR Analysis

^1^H-NMR spectra were recorded at 70 °C on a Bruker Ascend (11.75 T) Advance III HD 500 MHz spectrometer equipped with Bruker cryoprobe CP TCI 500S1 H-C/N-D-05-Z, automatic tunning matching, and autosampler. Chitosan samples were dissolved in a 0.3% (*v*/*v*) solution of DCl/D_2_O as described in ASTM standard F2260-18 [19]. The DA was determined from Equations (1)–(3)

(1) $$\left[\frac{H_{1}D+H_{2}D}{2}\right]=Deacetylated\;units\;proportion\;\left(D\right)$$

(2) $$\left[\frac{H_{1}A+\frac{H_{7}A}{3}}{2}\right]=Acetylated\;units\;proportion\;\left(A\right)$$

(3) $$\left[\frac{A}{D+A}\right]\times 100=Degree\;of\;acetylation\;\left(DA\right)\;\left(\%\right)$$

where H_1_D, H_2_D, H_1_A and H_7_A are the areas of the ^1^H NMR signals at 4.87 ppm, 3.20 ppm, 4.55 ppm and 2.01 ppm, respectively.

#### 2.2.2. Analysis of the Acetylation Reaction by FT-IR-ATR

FT-IR-ATR spectra were recorded on a Perkin Elmer Frontier spectrophotometer in the region of 4000–400 cm^−1^, with 16 scans and a resolution of 4 cm^−1^. Samples were in powder form and well dried.

#### 2.2.3. Determination of the Molecular Weight (MW)

The molecular weights of chitosan samples were determined by gel permeation chromatography (GPC) using a Viscotek system that consisted of a VE7510 GPC degasser, VE1122 solvent delivery system, a VE3580 RI refractive index detector and equipped with a NOVEMA Max analytical linear XL column. Pullulans with M_w_ from 10 to 2560 kDa were used as standards for the calibration curve. The software OmniSEC 4.6.2 was used for data processing. The eluent and solvent for chitosan was a buffer solution of 0.2 M HOAc/0.1 M NaOAc (pH = 4.5), flow rate was set at 0.5 mL/min and the column oven temperature at 35 °C. Before injection, samples were filtered with a syringe filter by Millex (0.45 µm) as is described by ASTM F2602 methodology [21].

### 2.3. Acetylation of Chitosan with Acetic Anhydride

Chitosan was dissolved in 10% (*v*/*v*) acetic acid to reach a 0.6 g/mL concentration. A desired amount of Ac_2_O was added, expressed as a molar relation to glucosamine (GlcN) units in the chitosan sample. The reaction mixture was prepared in a vial at room temperature, with stirring for 5 h. After that time, the solution was dialyzed, with a membrane of pore size 3.5–5 kDa against deionized water for a couple of days and then the solution inside the membrane was freeze-dried. The re-dissolved sample was treated with 5% methanolic KOH at room temperature for 5 h for O-deacetylation. Finally, the product was washed with methanol and water, and freeze-dried again.

### 2.4. Reduction of Chitosan Molecular Weight (MW)

#### 2.4.1. Chitosan MW Reduction by Hydrolysis in Aqueous Acidic Media

Chitosan was dissolved in an aqueous buffer solution of pH 4.5 (0.2 M HOAc/0.1 M NaOAc) to form a 1.5 mg/mL solution. The solution was divided into three portions that were kept at 4 °C, 20 °C and 50 °C for 42 days. Aliquots were taken from each portion every seven days and analyzed by GPC for MW determination.

#### 2.4.2. Chitosan MW Reduction by Ultrasound

**Figure 2:**
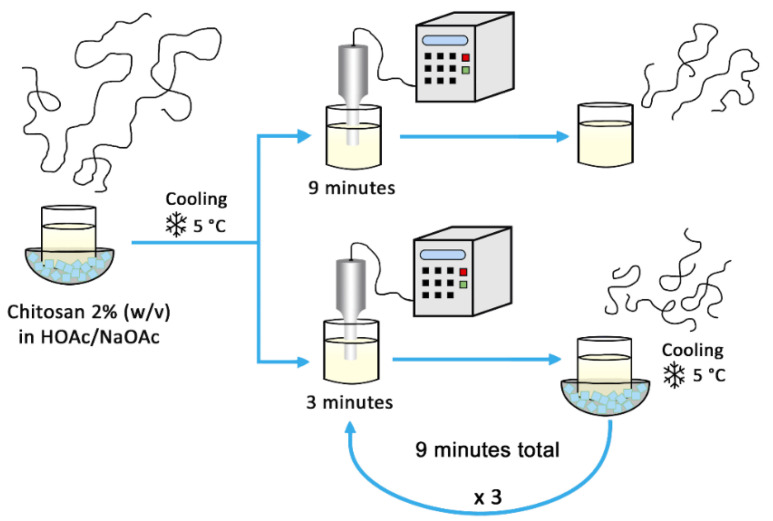
MW reduction with ultrasound.

A 2% chitosan solution (*w*/*v*) was prepared as above with the same buffer (pH = 4.5) and chilled in an ice bath to 5 °C. One portion of the solution was exposed for 9 min to ultrasound produced by a Sonics sonifier VCX 750 (frequency of 20 kHz) with a Cv334 sonotrode (model 630-0219, probe diameter = 13 mm, length = 139 mm, with an amplitude of 40%). The probe was immersed in the sample up to 15 mm from the bottom of the reactor (diameter = 49.2 mm, height = 66 mm). The other portion of the chitosan solution was exposed to ultrasound for 3 min, followed by cooling and stirring in an ice bath (for 15 min, until it reached 5 °C), the sample was then exposed to ultrasound again for 3 more minutes, cooled down and, finally, it was exposed for 3 more minutes to ultrasound. In this way, both chitosan samples were exposed to ultrasound for a total of 9 min, as the scheme in Figure 2 shows. MWs were determined by GPC.

## 3. Results and Discussion

### 3.1. Chitosan Characterization

The standard test for determining the degree of N-acetylation of chitosan by ^1^H-NMR from the ASTM (F2260-18) was used. This method is valid for chitosan soluble samples in the range of DA from 0 to 50% [19]. Samples were analyzed at 70 °C to reduce their viscosity and change the chemical shift of the solvent (H_2_O). The proton assignment can be observed in Table 1.

**Figure 3:**
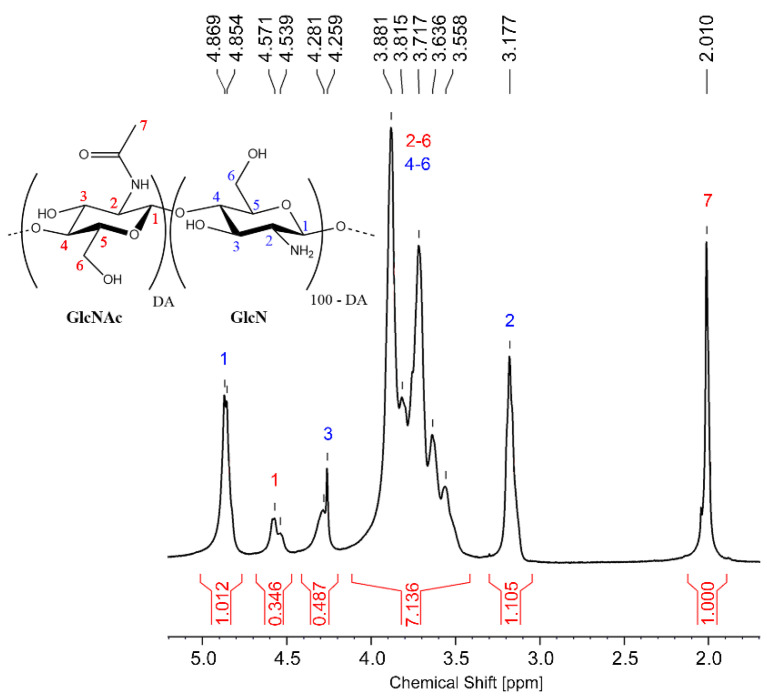
^1^H-NMR spectra of chitosan Q24 in DCl/D_2_O at 70 °C (500 MHz).

Due to the solvent’s signal overlap with H3, as can be observed in Figure 3 at 4.28 ppm, it was not considered for DA calculation. In this figure, it is easy to see the signal at 4.85 ppm, which corresponds to the anomeric proton of the deacetylated units, and the signal at 4.56 ppm, that is due to the anomeric proton of the acetylated units. The protons of the carbon next to the amine group in the deacetylated units appear at 3.16 ppm and the signals from 3.56 to 3.87 ppm correspond to the protons in the ring, in both the acetylated and deacetylated units. Finally, the protons of the methyl groups of the acetylated units appear at 2.01 ppm (more protected).

The standard test, ASTM F2602-18, for the determination of the MW of chitosan by GPC was used [21]. First, a calibration curve was made with pullulan standards in the range of 10 to 2500 kDa (PSS, Polymer Standard Service). The resulting weight average-MW (M_w_), number average-MW (M_n_) and peak-MW (M_p_) of chitosan Q-24 and Q10 are shown in Table 2. Additionally, shown are the corresponding averages of the degrees of polymerization and the polydispersity indexes for chitosans Q24 and Q10.

### 3.2. Chitosan Acetylation

Chitosan acetylation has been performed with different reagents, such as acetyl chloride in ionic liquids [22,23]; however, acetic anhydride has been used the most and usually reacted with chitosan in aqueous acetic acid solutions [24,25,26] (and in mixtures with alcohols and bases [27,28,29,30,31,32,33,34]), at room temperature and for several hours. It has been claimed that the presence of the alcohol in the media aids the reaction of the free amino groups in chitosan with long aliphatic chain anhydrides, and the selectivity of N-acetylation instead of O-acetylation. There is evidence that the reaction of chitosan with acetic anhydride can result in chains with a wide range of DAs, even up to 100% acetylation, with high reaction yields (85 to 95% of the recovered mass of the product [31]).

In this work, a homogeneous chitosan acetylation reaction was performed with acetic anhydride in a 10% (*v*/*v*) HOAc aqueous solution. Chitosans with DA of 24.1% (Q24) and 10.66% (Q10), were reacted with molar ratios of acetic anhydride/glucosamine units (Ac_2_O/GlcN) that ranged from 1 to 25. Only glucosamine units were considered for the ratio calculation since they have the free amino groups that can react with the anhydride. It should be noted that each glucosamine unit can react with up to three acetic anhydride molecules (through one amino and two hydroxyl groups), on the other hand, N-acetylated glucosamine repeat units can react with up to two anhydrides. Taking this into account, a chitosan sample with a DA of ~10% requires an estimated 2.89 moles of acetic anhydride per mole of repeat unit in order to react with all amino and hydroxyl groups in the polymer.

**Figure 4:**
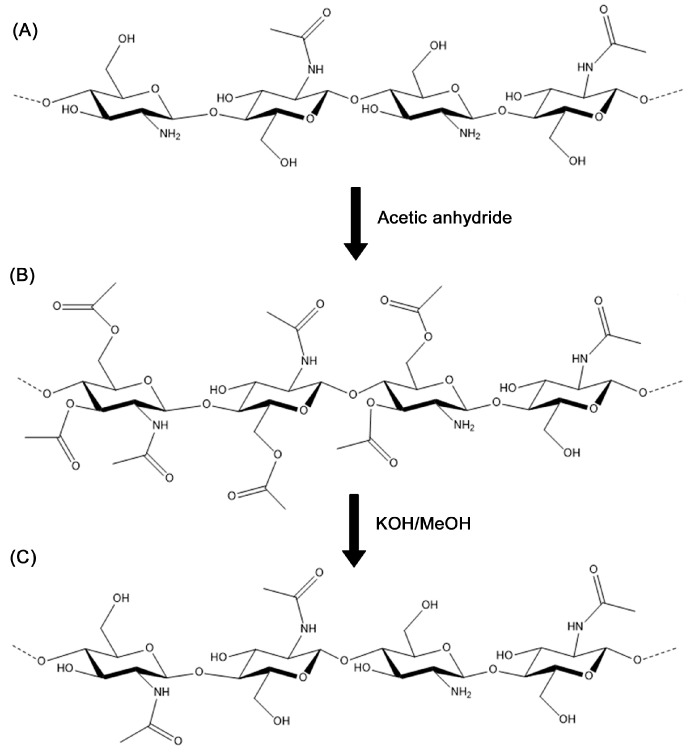
Acetylation reaction showing chitosan (**A**), O-acetylated and N-acetylated chitosan (**B**) and N-acetylated chitosan (**C**).

At the reaction conditions used, both the hydroxyl groups and the free amino groups in chitosan react (Figure 4A) and, therefore, the product of esterification and amidation is obtained (Figure 4B) [35,36]. The ester groups were then selectively hydrolyzed by alkaline treatment with 5% KOH (*w/v*) methanolic solution [24,25,37]. The final product is an N-acetylated chitosan (Figure 4C). The reaction yields obtained were above 98%.

**Figure 5:**
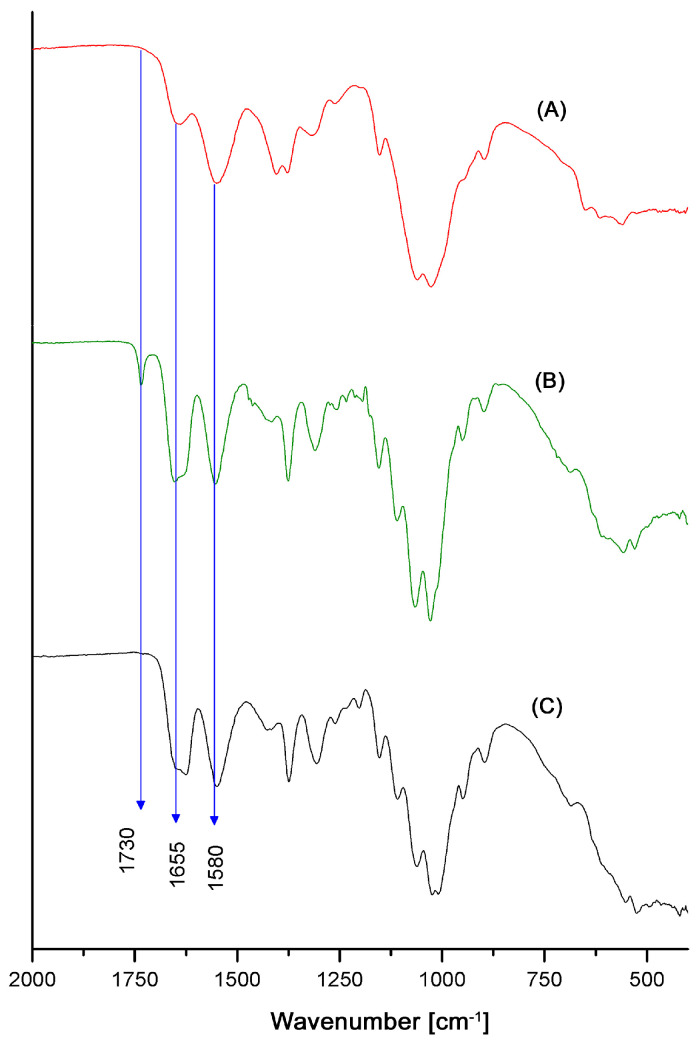
FT-IR-ATR spectra for chitosan Q24 (**A**), O-acetylated and N-acetylated chitosan (**B**) and N-acetylated chitosan (**C**).

The reaction pathway shown in Figure 4 was verified qualitatively by IR spectroscopy as is shown in Figure 5. In this figure, FT-IR-ATR spectrum A corresponds to the initial substrate (chitosan Q24) and spectrum B corresponds to the fully acetylated product showing an increase in the intensity of the band at 1655 cm^−1^ (which corresponds to the vibrations of the C=O bond in the amide groups) due to the presence of more N-acetylated glucosamine units [38]. Spectrum B also shows a new band at 1730 cm^−1^ which is associated with the stretching of the C=O bond in esters (produced by O-acetylation). Spectrum C in Figure 5 evidences the selectivity of the hydrolysis of the ester groups with methanolic KOH by the disappearance of the ester C=O band, while the band for the amide C=O at 1655 cm^−1^ remains unchanged.

**Figure 6:**
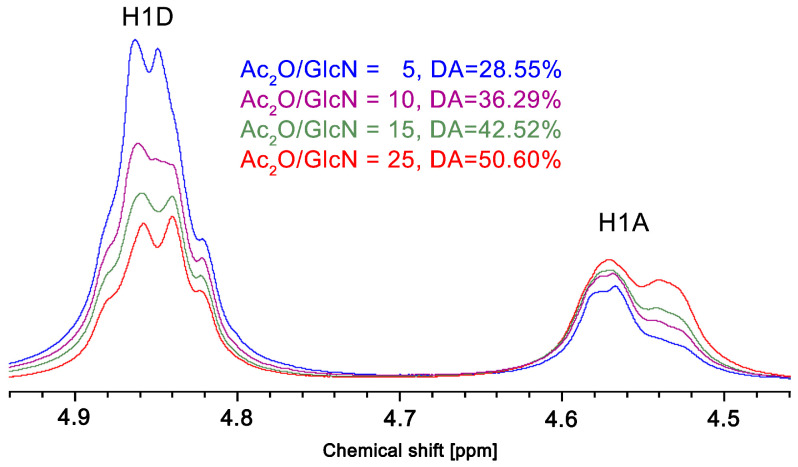
^1^H-NMR spectra of acetylated chitosans with different Ac_2_O/GlcN molar ratios (DCl/D_2_O, 70 °C, 500 MHz).

Figure 6 shows the ^1^H-NMR spectra of the products from the acetylation of Q10 with different ratios of Ac_2_O/GlcN. The peak labeled H1D that appears at 4.85 ppm corresponds to the anomeric hydrogen atom in the deacetylated repeat unit (GlcN), while the signal al 4.55 ppm (labeled H1A) is due to the anomeric hydrogen in the N-acetylated repeat unit. From the spectra shown, it is apparent that the intensities of the peak corresponding to the deacetylated unit (H1D) decrease as the Ac_2_O/GlcN ratio goes up and, at the same time, the peak of the acetylated unit (H1A) increases in intensity. This shows, as expected, that more N-acetylation occurs when more acetic anhydride is used.

Table 3 shows a summary of the results for the acetylation of chitosan with different Ac_2_O/GlcN molar ratios. Q10, which is a chitosan with 10% DA, required an Ac_2_O/GlcN ratio of around 25 to reach a DA of 50%, while, Q24 (chitosan with 24% DA) required less Ac_2_O to reach the same DA. It is not possible to determine accurately by NMR the DA for highly acetylated products (with DAs higher than 50%) since they become insoluble in the DCl/D_2_O solvent system required for NMR analysis. Ac_2_O/GlcN ratios higher than those shown in Table 3 produced insoluble chitosans that could not be characterized by ^1^H-NMR.

**Figure 7:**
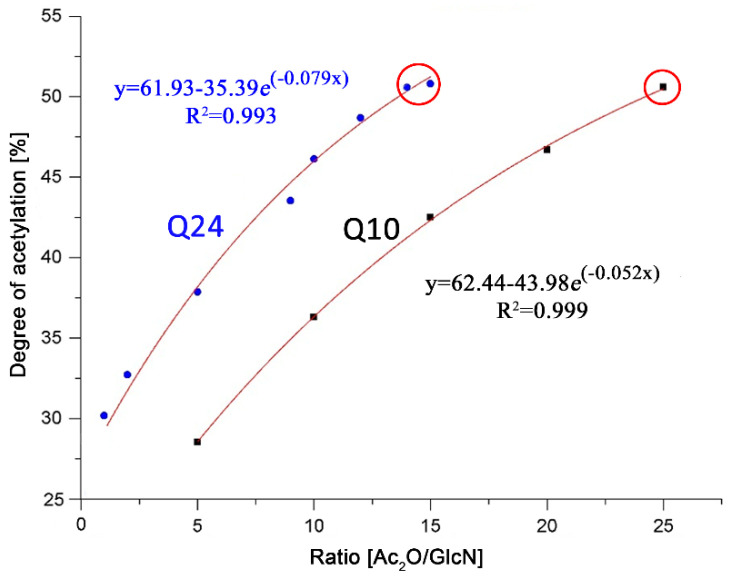
Relationship between the Ac_2_O/GlcN molar ratio and the DA of the acetylated chitosans Q10 and Q24 (data in circles correspond to partially insoluble products).

Plotted data from Table 3 and an exponential fitting curve are shown in Figure 7. The acetylated products from both chitosans show a similar trend, N-acetylation increases with a higher Ac_2_O/GlcN molar ratio. Higher ratios resulted in only partially soluble samples, probably due to DA’s higher than 50%, and, therefore, could not be determined reliably by NMR spectroscopy in the liquid state. Data in circles correspond to these partially insoluble products, which may show a DA that corresponds only to the soluble part. To test the fitting curve, an acetylation reaction with an Ac_2_O/GlcN ratio of 7.06 was performed on Q10, the product’s experimental DA from NMR resulted to be 33.18%, which is close to 32%, the expected value calculated from the fitting equation. This shows that the fitting could be used as a good reference to produce chitosans with any desired DA, as long as it is below 50% [39,40].

### 3.3. Chitosan MW Reduction by Hydrolysis in Aqueous Acidic Media

The reduction of the molecular weight of chitosan has been reported to occur with aqueous acidic solutions [41], enzymes [42], ultrasound exposure [43,44,45,46,47], nitrous acid [48,49], hydrogen peroxide [50,51] or heating with microwaves [52]. In the case of acidic hydrolysis, two reactions can occur, the cleavage of the main chain by hydrolysis of the glycosidic linkages through an S_N_1 reaction and the hydrolysis of the N-acetyl groups (through an S_N_2 reaction) [53,54]. It has been reported in the literature that a minimum of deacetylation can also occur during long periods of storage times [55].

**Figure 8:**
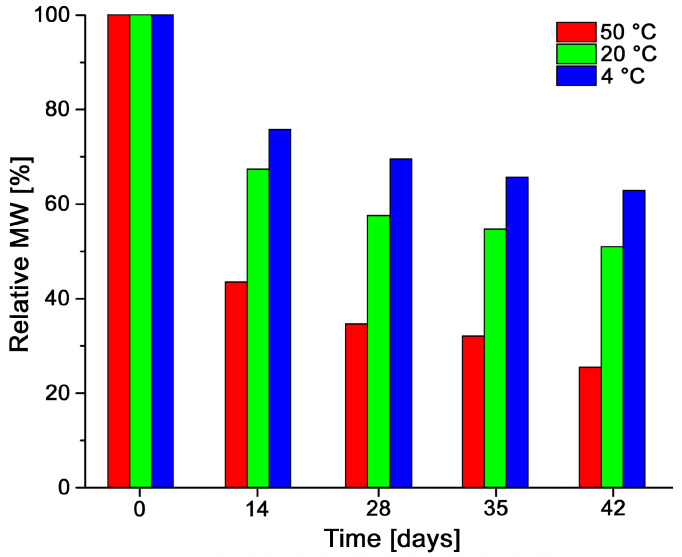
Reduction of the MW of chitosan Q24 at pH 4.5 at 4, 20 and 50 °C.

If done under control, aqueous acidic conditions can be a readily available and useful method to produce chitosans with shorter chains. For some applications, small chains may be preferred to avoid high solution viscosities, for example [53,56]. The shortening of chitosan chains at pH 4.5 was evaluated as a function of temperature and time, and determined by GPC. Figure 8 shows the relative decrease in MW (weight-average molecular weights) of Q24 at 4 °C, 20 °C and 50 °C during 42 days. It can be observed that, during the first 14 days, the initial MW of 1583 kDa was reduced significantly more at 50 °C (690.9 kDa, 56.5% MW reduction) than at 20 °C (1070 kDa, 32.6% MW reduction) or at 4 °C (1203 kDa, 24.2% MW reduction). This is expected since the degradation reaction requires energy to break bonds in the chitosan chains and therefore is more favorable at higher temperatures, similar results have been reported [53]. For the second 14-day period, the MW of the sample kept at 50 °C decreased in 65.4% while at 4 °C the reduction was only 30.3% of the MW. After 42 days of hydrolysis, the chitosan kept at 50 °C lost a total of 74.5% of its size, while at 20 °C the loss was 48.9% and 37.1% for the sample at 4 °C. The yield of acidic hydrolysis was 83.2%. Gordon et al., have reported similar results for the degradation of chitosan after storage for two weeks, 1, 3 and 6 months at 4, 25 and 40 °C. For six months at 40 °C, they observed a final MW of 64 kDa, which corresponded to an 85% reduction [41].

**Figure 9:**
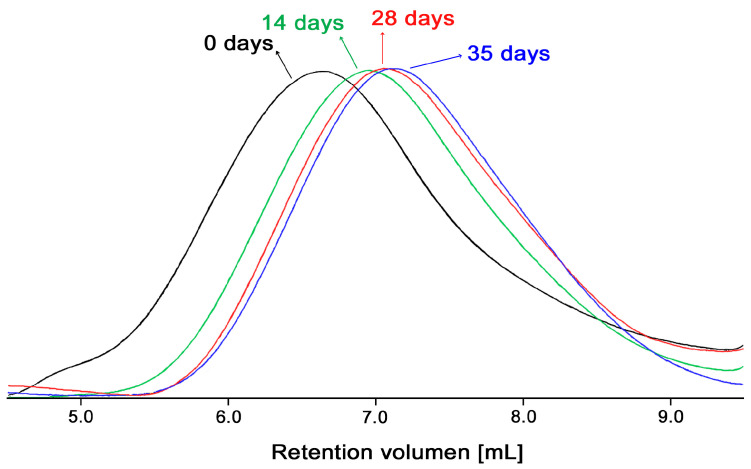
GPC chromatograms of chitosan stored at 50 °C for 0, 14, 28 and 35 days.

Figure 9 shows the GPC chromatograms of chitosan Q24 stored at 50 °C after 14, 28 and 35 days, the chromatogram for Q24 is also shown as a reference. A displacement of the retention volumes (from 6.613 mL to 6.938 mL, 7.038 mL, 7.108 mL, respectively) can be observed, this evidences a reduction of the hydrodynamic volume of the polymer chains which is related to the decrease of the MW.

### 3.4. Chitosan MW Reduction by Ultrasound

There is a consensus that when exposed to low frequency ultrasound, chain cleavage is mainly due to mechanical forces produced during cavitation [45,57] and it has been reported to be a fast method to reduce its MW [46,47,58]. Interestingly, Baxter et al. and Wu et al. have reported that the degradation by ultrasound does not affect the DA of chitosan when short periods of ultrasound exposure are used [45,58]. However, there is evidence in the literature that chitosan can be deacetylated considerably when treated extensively with ultrasound, for a long period of time and in strong alkaline conditions [59,60].

**Figure 10:**
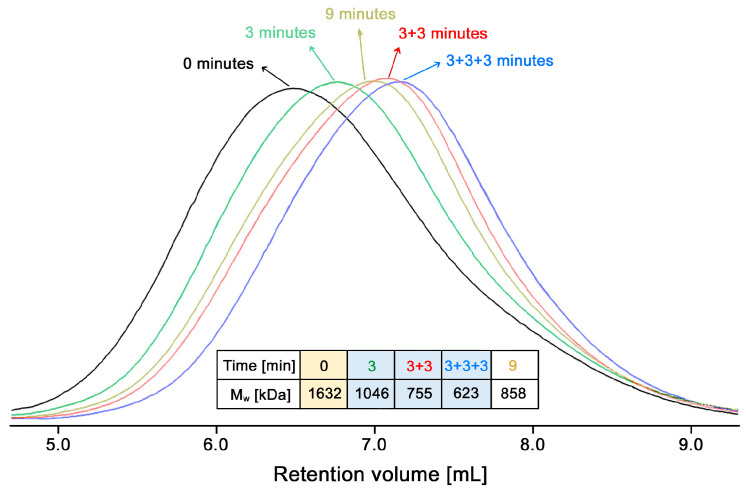
GPC chromatograms of chitosan treated with ultrasound for 9 min straight and for a sequence of three 3-min treatments.

Figure 10 compares the GPC chromatograms for the initial chitosan, chitosan samples treated with ultrasound for one, two and three periods of 3 min each, and also a sample treated continuously for 9 min. The data attached to the figure corresponds to the weight-average MW obtained from the distribution for each product with the GPC software. The MW of all samples was reduced significantly with ultrasound treatment. As expected, more exposures (of 3 min each) resulted in lower MWs. However, it is observed that the continuous exposure for 9 min to ultrasound is less efficient than the three 3-min exposures for reducing the MW of chitosan. In the first case, longer chitosan chains, 858 kDa, were obtained, compared to chains of 623 kDa, for the second case. This may have to do with different factors, such as the homogenization of the sample by stirring in between the 3-min treatments. Due to the high viscosity of some chitosan solutions, the effect of the ultrasound waves may not be homogeneous across the entire volume of the solution, the portion close to the probe is exposed to a different intensity than a portion of the solution further away. Evidence of this observation is that the GPC chromatogram for the sample that was treated continuously for 9 min shows a greater peak width at half-height (1.63 mL) and wider PDI (4.3) than the intermittent treatment (three 3-min treatments with cooling and stirring in between), which shows 1.58 mL half-height width and a PDI of 3.8. Both parameters show that there is a larger variety of chain lengths for the former case. It should be noted that the GPC chromatograms in Figure 10 do not show a bimodal distribution such as Trzcinski et al. observed [46]. These authors explained that a bimodal distribution could be explained by a midpoint chain cleavage model, on the other hand, the random scission of chains model would produce unimodal distribution, as presented by Wu et al. [45].

In addition to this, exposure to ultrasound is usually accompanied by heat transfer. It was found that ultrasound could increase the temperature of the sample up to 13.6 ± 0.9 °C/min. Taking into account the results reported by Wu et al. [45] showing that chain scission by ultrasound is greater at low temperatures, samples were immersed in an ice bath and stirred after each ultrasound exposure for a more efficient method.

**Figure 11:**
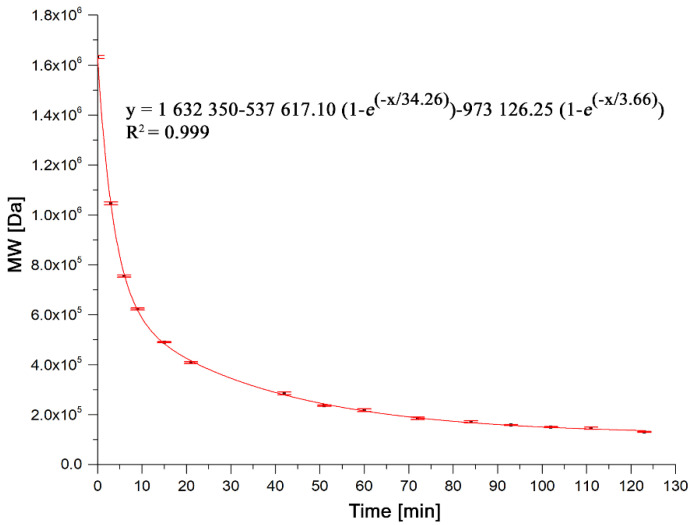
Reduction of chitosan MW with ultrasound (20 kHz) exposure time.

The MW of chitosan samples treated with ultrasound for up to 123 min (forty-one 3-min treatments) is presented in Figure 11. As can be seen, ultrasound treatment applied to a high MW chitosan in solution produced a rapid shortening of the chains and is dependent on the exposure time to ultrasound, as expected. The rate of degradation is high and corresponds to that of a chitosan solution concentration well above the overlap and entanglement concentrations of a high MW chitosan, as explained by Wu et al. [45]. An exponential equation that roughly fits the data can be used to estimate the treatment time needed to produce any desired MW. The lowest MW obtained was 131 kDa, a 92% reduction in MW of the original polymer. Similar graphs have been reported by Popa-Nita et al. and also Wu et al. [45,47], each for different experimental conditions.

The results show that ultrasound is a more efficient method to reduce the MW of chitosan than the acidic hydrolysis in terms of time. For example, to reduce the MW of chitosan to 25% of its original size, hydrolysis for 42 days at 50 °C is required, compared to 40 min of ultrasound exposure time.

An advantage of the reduction of the MW with ultrasound is that this procedure does not affect the composition (DA) of the chitosan sample. To test this, a chitosan sample with DA = 27.46% that was exposed to ultrasound for three 3-min periods resulted in a product with DA = 27.26%. Further treatment for three more periods of 3 min each resulted in a chitosan with a DA of 27.62%. No change in DA by ultrasound has also been reported by Wu et al. [45]. However, Muzzarelli et al. have reported that chitosan deacetylation occurs with long continuous ultrasound exposures (40 min) in alkaline media [59].

**Figure 12:**
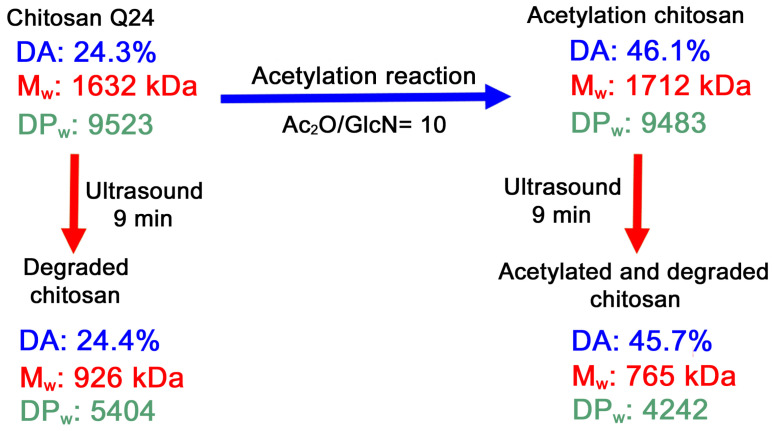
Chitosan Q24 acetylation and degradation scheme.

Finally, as an example of the versatility of the methods studied, Figure 12 shows a scheme in which chitosan Q24 (DA 24.3%, 1632 kDa, DP_w_ 9523) is transformed into three other products, a more acetylated chitosan (DA 46.1%) with roughly the same DP_w_ (9483), a chitosan with the same DA but with lower MW (DP_w_ 5404) and a chitosan with a higher DA and lower MW and DP_w_ (DA 45.7%, 765 kDa, 4242). This simple approach to control the DA and MW of chitosan could serve as a starting point to industrially synthesize tailor-made products and reference materials.

## 4. Conclusions

Many of the applications of chitosan depend on its chemical structure: the degree of N-acetylation and molecular weight. Therefore, it is important not only to fully characterize the samples but to develop methods to synthesize chitosans with any specific DA and MW. In this regard, acetic anhydride can be used as an acetylation agent in a mild, simple and high-yield reaction that can be easily controlled by the molar ratio of the reagent to glucosamine repeat units in the substrate. This acetylation methodology does not affect the size of the chitosan chains. On the other hand, MW reduction can be achieved in two simple ways, by aqueous acidic hydrolysis or by ultrasound. Both depend on the treatment time, the former is simpler and requires no special equipment but the kinetics are slow, the latter is more efficient, and does not change the DA. In this way, tailor-made chitosans, with any desired MW and DA, can be produced from samples with high MW and low DA.

Being able to produce chitosans with specific structures (DA and MW) could aid to interpret contradictory data that can be found in the literature regarding the polymer’s properties and characteristics. It may also contribute to the making of chitosan standard samples for easier characterization of this promising biopolymer and to study the behavior of mixtures of chitosans with different DA or MW.

## Figures and Tables

**Table 1:** ^1^H-NMR chemical shifts of chitosan signals.

Proton	δ (ppm)	
	GlcN (D)	GlcNAc (A)
H1	4.84–4.86 (doublet)	4.54–4.58 (doublet)
H2	3.15–3.19 (triplet)	3.56–3.87 (multiplet)
H3	4.22–4.28 (doublet)	
H4–H6	3.56–3.87 (multiplet)	
H7	*–*	2.01 (singlet)

**Table 2:** MWs and DPs of chitosan Q24 and Q10, by GPC.

Chitosan	M_w_ (kDa)	M_n_ (kDa)	DP_n_	M_p_ (kDa)	PDI ^1^
Q24	1632.0	233.6	1363	582.6	7.221
Q10	1206.4	259.4	1566	455.7	4.667

^1^ PDI = Polydispersity index (M_w_/M_n_).

**Table 3:** DA of acetylated chitosans with different Ac_2_O/GlcN molar ratios.

Ac_2_O/GlcN	Chitosan Q24	Chitosan Q10
	DA (%) ^1^	DA (%) ^1^
0	24.31 ^2^	10.66 ^2^
1	30.17	-
2	32.71	-
5	37.87	28.55
9	43.53	-
10	46.15	36.29
12	48.69	-
14	50.47 ^3^	-
15	50.63 ^3^	42.52
20	-	46.69
25	-	50.60 ^3^

^1^ DA determined by ^1^H-RMN, ^2^ Initial chitosan Q10 and Q24, ^3^ Partially insoluble product in acidic media.

## Data Availability

The data presented in this study are available on request from the corresponding author.
